# Time dynamics of COVID-19

**DOI:** 10.1038/s41598-020-77709-4

**Published:** 2020-12-03

**Authors:** Cody Carroll, Satarupa Bhattacharjee, Yaqing Chen, Paromita Dubey, Jianing Fan, Álvaro Gajardo, Xiner Zhou, Hans-Georg Müller, Jane-Ling Wang

**Affiliations:** 1 Department of Statistics, University of California, Davis, Davis, CA 95616 USA; 2 Department of Statistics, Stanford University, Stanford, CA 94305 USA

**Keywords:** Scientific data, Statistics, Epidemiology

## Abstract

We apply tools from functional data analysis to model cumulative trajectories of COVID-19 cases across countries, establishing a framework for quantifying and comparing cases and deaths across countries longitudinally. It emerges that a country’s trajectory during an initial first month “priming period” largely determines how the situation unfolds subsequently. We also propose a method for forecasting case counts, which takes advantage of the common, latent information in the entire sample of curves, instead of just the history of a single country. Our framework facilitates to quantify the effects of demographic covariates and social mobility on doubling rates and case fatality rates through a time-varying regression model. Decreased workplace mobility is associated with lower doubling rates with a roughly 2 week delay, and case fatality rates exhibit a positive feedback pattern.

## Introduction

As of May 1, 2020, more than 3 million cases of COVID-19 had been reported worldwide, leading to more than 200,000 coronavirus related deaths^1^. The World Health Organization declared the situation a pandemic on March 11, 2020, and nearly all countries have been exposed to SARS-CoV-2, the betacoronavirus which causes the disease^2^. Despite the far reach of the virus, the pattern and rate of its spread within a population is not uniform. Some countries like the US have seen marked increases in case and death counts per capita, even after implementing distancing measures, while others like Japan have been able to keep the spread of disease low for long durations despite comparatively lax social restrictions. Measures that mitigate spread in one case may not work uniformly across countries due to effects of demographics and timing, among other factors. Data-driven analyses of the time-dynamics of cases and deaths are of central importance to characterize underlying forces and unexplained variation.

Global efforts to “flatten the curve” of COVID-19 cases translate quantitatively to decreasing epidemiological statistics like doubling rates via social distancing campaigns, improved hygiene and case tracking. Early statistical inquiries have focused on estimation of doubling rates, and case fatality rates with SIRD and SEIM models^3,4^, which are compartmental epidemiological models. Such models have been useful in determining whether or not disease-free equilibrium, that is, eradication, is stable and attainable in the context of other contagious diseases like cholera^5^. Other quantitative approaches have included forecasting the number of cases worldwide using time series analysis modeling^6^ or quantifying the effects of prevention efforts like social distancing^7,8^, public gathering, and travel restrictions^9,10^ for single countries.

Processes that grow exponentially, such as the case load of unmitigated COVID-19 transmissions are characterized by a fixed doubling time. In reality, however, the doubling time is a dynamic quantity, which changes continuously due to mitigation efforts and the inherently changing nature of virus-spreading mechanisms. It is then vital that policymakers and researchers have access to frequent and up-to-date estimates of doubling time^11^. For example, Du et al.^12^ provided early, fixed-in-time estimates of epidemic parameters of COVID-19 (e.g. growth rate, doubling time, basic reproduction number, case detection rate) during the first 50 days of onset in China. In recent work^13,14^ the basic reproduction number and doubling time have been studied in a dynamic manner by considering a varying coefficient model with daily new cases as the response and time as a predictor. A related approach focused on the real-time estimation of case fatality rates using Poisson mixture models^15^. Our analysis complements these studies and introduces an alternative way of obtaining relevant dynamic quantities, associating metrics of disease progression with baseline covariates across many countries. Recent research has queried the effects of population age, temperature, humidity^16,17^, lockdown interventions^9,10^, community mobility patterns^18,19^, and other factors on the spread of the virus. Modeling the transmission dynamics of COVID-19 based on predictors is key to understanding the impact of distancing practices and other policies on spread mitigation and prevention. We refer to^20^ for a more thorough review of recent epidemiological analyses.

It should be noted that COVID-19 analyses based on published case and death counts, including those conducted here, are subject to the same biases which affect the accuracy of the data, primarily due to under-reporting^21^, the degree of which varies by country^22^. The reasons for such under-reporting are many, including insufficient testing materials, political incentives, and administrative delays. With this caveat in mind, the study of available data may nevertheless provide useful insights and stimulate further research, aided by the statistical methodology that we present in this paper.

Specifically, we propose functional data analysis as a tool for analyzing the time-dynamics of COVID-19 as quantified by case and death numbers across countries. Functional data analysis (FDA)^23–25^ aims to detect structures and patterns in samples of random trajectories through functional principal component analysis^26,27^, empirical dynamics^28^ and other methods, where entire curves are viewed as data atoms. This methodology is uniquely suited for the analysis of COVID-19 data since the cumulative case counts across countries amount to a sample of random curves observed over time.

FDA based approaches enjoy an added strength in that they leverage the latent information shared between countries to boost the efficiency of predictions and facilitate comparisons of the trajectories across countries. We use FDA methodology to study patterns of growth for case counts and to quantify the performance of various countries as the pandemic progresses. We also showcase an approach for predicting future case counts which may be used to understand whether a country is doing better or worse than expected. Finally, we explore the effect of variables like population density, demographic age structure, and mobility reduction efforts on total case trajectories as they vary over time. Throughout, our focus is on associations which may suggest but do not establish causality.

## Results

### COVID-19 dynamics across countries

#### Main patterns of disease propagation

**Figure 1:**
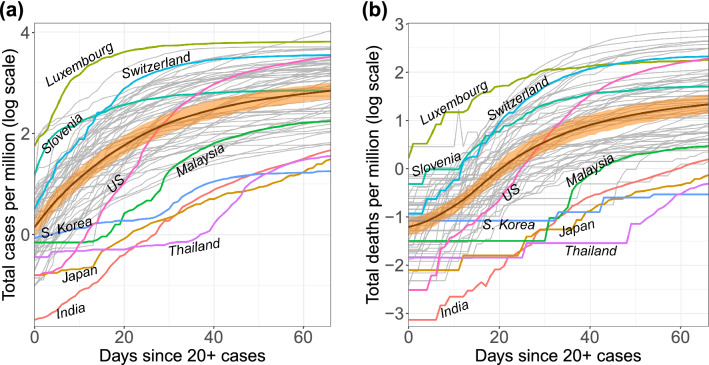
Trajectories of **a** total case count and **b** total death count per million individuals on log scale. The time window spans the 67 days since at least 20 confirmed cases were reported. Smoothed mean curves are marked by bold black lines. The orange ribbons represent pointwise 95% bootstrap confidence bands for the overall mean functions.

The cumulative COVID-19 case and death count trajectories per million people (in log scale) for 64 countries are displayed in Fig. 1 (see “Methods”). The trajectories are shown for a 67-day interval after the first time a country reports at least 20 confirmed cases, which is taken as the origin of the time domain and thus corresponds to different calendar times (see “Data”).

**Figure 2:**
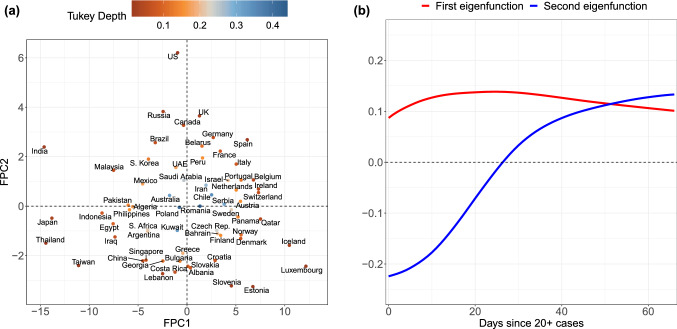
Visualizing (**a**) the Functional Principal Component (FPC)-space representation of case trajectories, where coordinates represent the amount of deviation from the mean curve in the direction of (**b**) the first (red) and second (blue) eigenfunctions. The two-dimensional representation in FPC space captures 97% of the variation in the sample of case load trajectories across countries. Points are colored according to their Tukey depth, where higher depths indicate trajectories closer to the mean curve.

For the 64 countries in the study, case counts per million generally follow one of four paths over time. They are either (1) consistently higher than average (e.g., Switzerland), (2) consistently lower than average (e.g., India), (3) initially lower but then experience a dramatic increase over time (e.g., the US), or (4) initially higher before entering a period of control (e.g. Slovenia). These archetypes are derived from the extreme ends of the two main modes of variation^29,30^ observed for the sample, which emerge from functional principal component analysis (FPCA, Fig. 2). FPCA is similar to ordinary principal component analysis in the sense that it projects high dimensional curve data into a low dimensional space, representing them as a random vector of functional principal component (FPC) scores, as seen in Fig. 2a (see “Methods”). These scores are matched with smooth *eigen* *functions*, displayed in Fig. 2b, which reveal the primary patterns of variation in the sample of functional objects. For the case load trajectories, a 2-dimensional representation was found to adequately represent the sample of curves.

An inspection of the eigenfunctions and their corresponding FPC scores reveals which of the four patterns a country generally follows (Fig. 2). FPC scores of countries with consistently higher case trajectories are located in the right half-plane, and those with uniformly lower rates in the left. Analogously, countries which experience a dramatic increase in cases per million lie in the upper half of the plane, and those which successfully slow their spread in the lower half. Countries which follow similar trajectories are clustered together in FPC space, with outliers located on the outskirts of the point cloud. While some outliers are apparent from a simple visual inspection of the original curves (e.g. Luxembourg, Thailand, India and Japan), other atypically-shaped trajectories are initially obscured in the crowd of curves until revealed in FPC space (e.g. the US).

**Figure 3:**
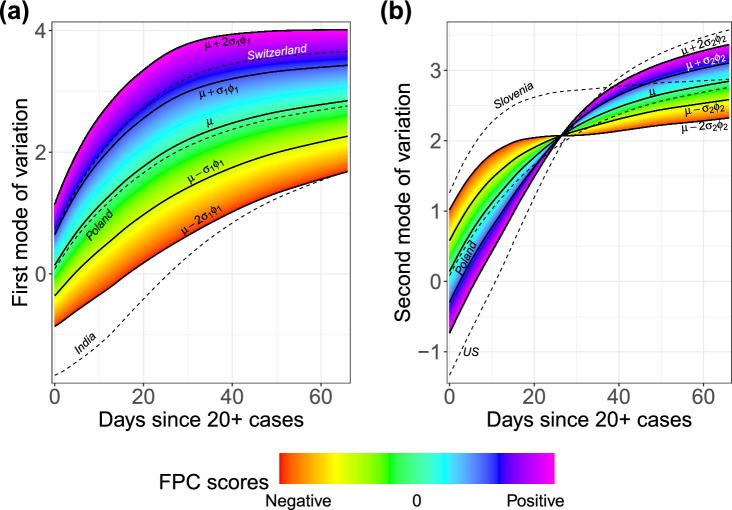
The (**a**) first and (**b**) second modes of variation (solid lines) based on mean function $\mu$ and first and second eigenfunctions $\phi _1, \phi _2$, respectively, for the total cases process. The sample standard deviations of the first and second FPC scores are $\sigma _1=5.77$ and $\sigma _2=1.96$ respectively. The dashed lines depict fitted trajectories for select countries which are extreme in either mode and Poland which exhibits a trajectory close to the mean function.

The concept of modes of variation is useful for visualizing the range of FPC scores as a spectrum of curves (Fig. 3). For example, India’s deviation from the mean curve is largely explained by just the first mode of variation. Its very negative first FPC score places it far below the mean throughout the entire interval. In this sense the first FPC score is similar to a random intercept in a linear mixed model, since the first eigenfunction is roughly constant over time. Incidentally, the slight curvature exhibited in this eigenfunction allows for flexible modeling of the “curve bending” phenomenon as demonstrated by Switzerland, which has a very positive first FPC score.

The US departs from the typical mean trajectory in a different way: Its case count per capita has increased dramatically by the end of the time interval, which is seen by its very positive second FPC score. The second mode of variation captures a subtle curvature that would be missed in parametric modeling. In this sense the second FPC score modulates the shape of the curve rather than its magnitude. Nearly all cumulative case trajectories begin to level off after roughly $t=25$ days, a trend which is illustrated neatly by the inflection point in the second mode of variation. One may then interpret the first month of exposure as a sort of “priming period,” after which (non-outlier) trajectories exhibit more stable trends. This interpretation echoes the findings of^31^, who also identify the first few weeks of exposure as a critical time for disease management.

Countries which are clustered together in FPC space may also share other underlying structures such as geographic proximity, exemplified by the Western European countries in the upper-right quadrant of Fig. 2. In some cases, far-flung places may act as if they are part of a distinct geographic cluster, such as Panama and Qatar with trajectories that are very similar to those in the Scandinavian block.

Countries’ performances in the two main modes of variation can be evaluated by comparing their respective FPC scores. Higher scores indicate higher rates of spread for both modes of variation. For example, Switzerland has a much higher first FPC score than India, reflecting that the case count per capita is higher for Switzerland throughout the time period that we study here. Similarly, the case trajectory in the US is seen to have risen much more over time than that of Slovenia, say, since the second FPC score of the US is very large and positive while it is negative for Slovenia.

#### Comparisons via rank dynamics

**Figure 4:**
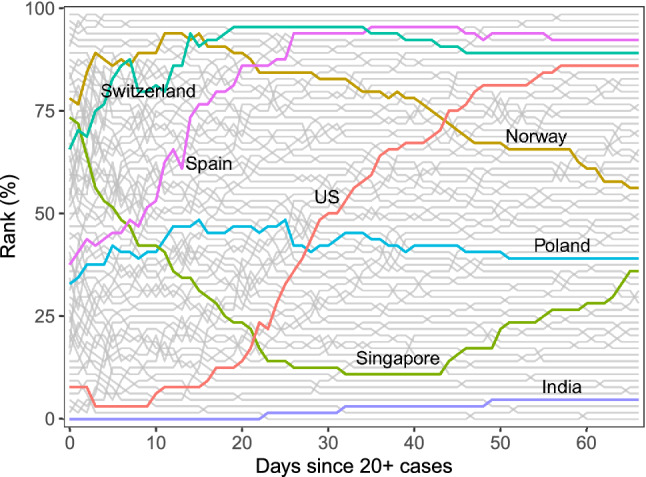
Rank trajectories during the first 67 days since exposure. Countries which appear higher in rank percentiles for a given time point have more cases per capita. Many intersections of rank trajectories correspond to periods of high volatility; fewer intersections indicate an more stability in performance across countries.

While FPC scores are useful for comparing and classifying country trajectories, they require multivariate comparisons, since each country is represented by two scores. It is useful to complement these comparisons by ranking each country by cumulative case counts per million, where higher percentile ranks correspond to increased infection rates and a generally worse situation. Ranking can be done at each fixed time and then analyzed with rank dynamics^32^. The percentile ranks and their time-evolution are illustrated in Fig. 4 with a few notable curves highlighted. Higher percentiles signify more cases per capita. Switzerland’s transmission rates have been among the most severe, while India has performed well consistently throughout the time period considered here. Both countries’ integrated ranks are among the highest and lowest, respectively, where integrated rank is the average rank over time. Both display low rank volatility (see Section S.6 in the Supplement), which is a measure of how ranks change over time, as their overall positions remain relatively stable throughout the time period.

In terms of ranks, the situations in Spain and the US have substantially worsened over time, which is in line with the FPCA results, where Spain and the US were found to have dramatically increasing case counts. These large shifts are reflected in their rank volatility, in which Spain and especially the US visibly stand out (Section S.6 in the Supplement). On the other hand, the epidemic situations in Norway and Singapore have improved over time relative to other countries, especially during the first 40 days for the latter. However, Norway still has a somewhat severe situation overall, and Singapore’s percentile rank starts to rise in the last third of the period. In contrast to these extremes, Poland exhibits a relatively stable rank over time and also a moderate epidemic situation when compared to other countries. Overall the percentile trajectories are more volatile during the first 25 days. By the end of the interval however, the ranks have become relatively stable, as seen by the fewer number of intersections between paths.

### Time evolution and forecasting of case trajectories

#### Frequently-updating forecasts for viral transmission

Since the virus reaches countries at staggered times, countries with earlier exposure times hold valuable information for predicting those which were more recently exposed. This makes it possible to employ a dynamic FPCA approach to make short-term predictions of trajectories by borrowing “future” information from countries for which a trajectory over a longer time interval is available, due to their earlier exposure in calendar time. Like FPCA, this is based on the assumption that there are common shape features that explain a majority of curve variation. Borrowing information from trajectories observed over a longer time stretch to inform trajectory prediction for those observed over a shorter time period is the key to the dynamic FPCA (dynFPCA) method.

Motivated by the rapidly updating nature of the COVID-19 case trajectories, dynFPCA harnesses the evolving trajectory data to predict case counts in the near future. We illustrate this for 10-day-ahead predictions that are constructed on repeating intervals. Then, after the 10-day forecast has passed, one can compare predictions with the observed trajectories. An observed case count which exceeds the prediction corresponds to worse than expected performance during that time window. Similarly an overestimate in prediction signifies better than expected containment of the virus.

Since FPCA summarizes an observed curve as a finite vector of FPC scores, it is enough to predict just the FPC scores for the curve we wish to forecast. The scores may then be translated back into a curve on the entire interval, even if the scores are only estimated using a partially observed trajectory (see “Methods”: dynFPCA). The FPC scores are estimated using the conditional expectation approach^33^, and the number of scores to estimate was chosen by an AIC criterion. The expression for conditional expectation depends on quantities which are estimated from the entire sample of curves and thus predictions gain strength by pooling all countries’ available information; see the discussion in “Methods” for technical details.

**Figure 5:**
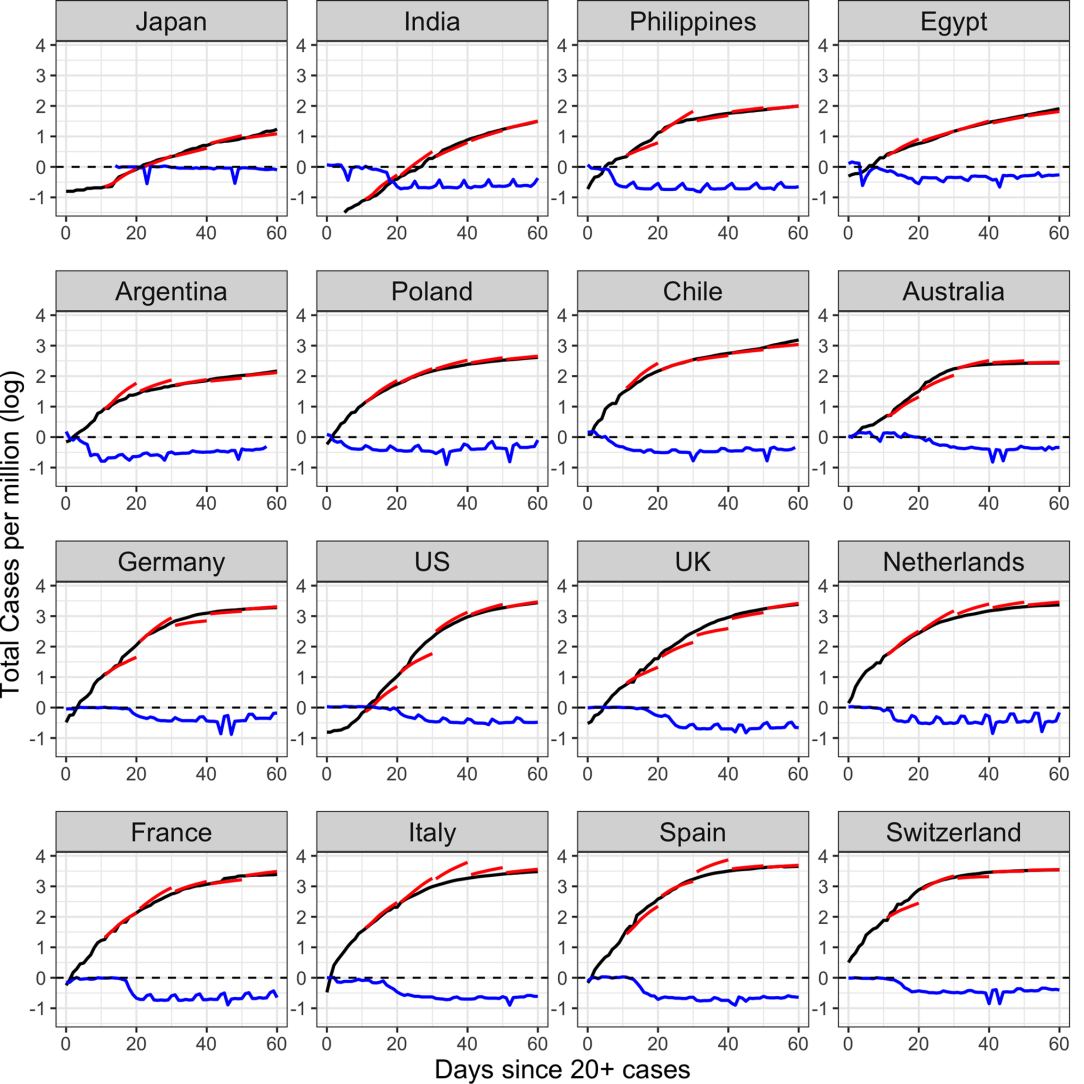
Forecasts of case counts for countries with early (top 8) and late (bottom 8) mobility reduction responses. The black curve represents the observed cumulative case counts, and the red piece-wise curves represent predictions for the 10 following days, made at 10, 20, 30, 40, and 50 days, respectively. The blue curve displays percent decrease in workplace mobility as measured by Google relative to a baseline index of mobility for a typical day before the onset of COVID-19 (“Methods”:“Data”). A day in which all mobility has stopped corresponds to −1 or a 100% reduction.

The predictions can be viewed in conjunction with the reduction in workplace mobility per country as reported by Google COVID-19 Community Mobility Reports^34^ to quantify the magnitude of voluntary or mandated lockdowns (see “Methods”: “Data”). Early studies of the effects of lockdowns have quantified the delay until case numbers are affected to be roughly 2 weeks^7,35^. Japan is a visible outlier in both measures (Fig. 5), in the sense that it has a very low and flat cumulative case curve, while mobility levels do not decrease much, as the Japanese government did not issue lockdown orders and employers by and large continued to require physical presence at the workplace. Other countries with relatively flat curves include India and the Philippines. These countries had major declines in workplace mobility early in the trajectory when the total caseload was still low.

Another group of countries with early declines in mobility includes Argentina, Chile, and other South American countries. For countries that reduced mobility early, our forecasts reach much lower heights than those which waited to restrict social gatherings. This latter group consists of mostly European countries (including Germany, the UK, the Netherlands, France, Italy, Spain and Switzerland), where reduced mobility occurred only after a sizeable number of cases had accumulated. At this point, the intervention efforts may have come too late to slow the momentum of infectious spread. As a consequence, the trajectories of cases for these countries not only increase more rapidly but also reach higher overall levels than predicted. After the first two 10-day periods, which is characterized by high volatility as seen in the rank analysis, the dynFPCA method tends to predict the trajectories quite accurately.

#### Time-dynamics for correlates of doubling rates and case fatalities

To quantify the effect of mobility reduction on dynamic FPCA predictions, we studied its effect on doubling rate, in addition to the effects of other demographic predictors that might play a role in shaping the trajectories. Understanding the factors which correlate with increased or decreased rates of infection is critically important for policymakers and societies. As local situations evolve and the pressure to reopen mounts, an equally important aspect is understanding how these associations might change over time, and when predictors are particularly relevant, as it is likely that the effect of predictors is not stationary but rather varies over the course of the pandemic.

**Figure 6:**
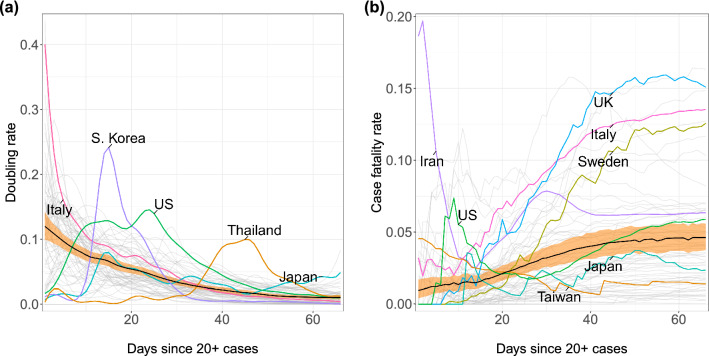
Time-varying (**a**) doubling rates and (**b**) case fatality rates for all countries. The mean function is depicted in black and the orange ribbon represents a pointwise 95% bootstrap confidence band.

To investigate the time-varying relationship between a country’s demographics, mobility reduction, and outcome measures such as doubling and case fatality rates, we applied a functional concurrent regression model and empirical dynamics (“Methods”). Doubling rate, $\gamma (t)$, and case fatality rate, CFR(*t*), as time-varying responses have been traditionally used in epidemiological modeling^20,31,36,37^ (see “Methods” for computational details). Low doubling rates indicate successful containment of viral transmissions, while low case fatality rates indicate better outcomes in terms of the mortality of infected populations. Mean doubling rates and case fatality rates over time are depicted in Fig. 6. We caution that estimates of the case fatality rate contain additional uncertainty, since reported cases are an undercount and not all deaths caused by COVID-19 may be correctly attributed to the infection.

**Figure 7:**
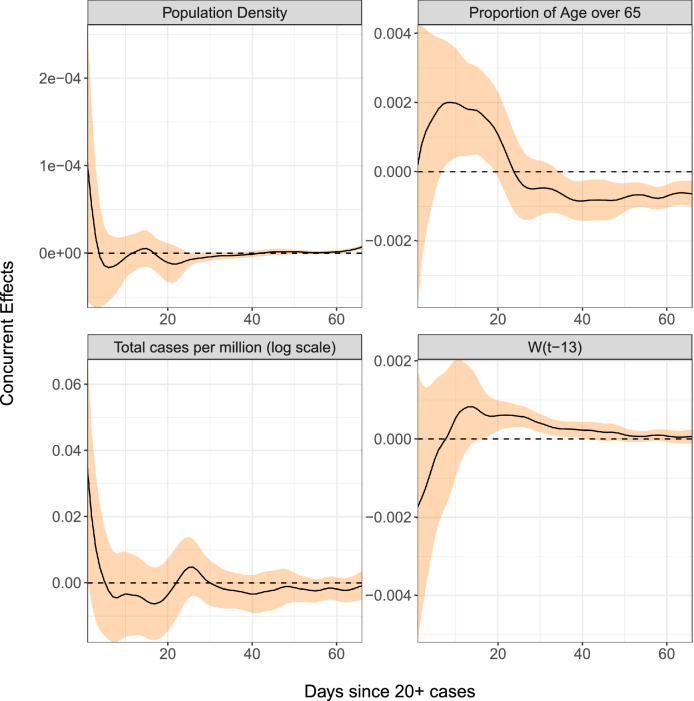
Time-varying regression effects of population density, the fraction of population over age 65, total cases per million and the percentage of the reduction from baseline in workplace mobility patterns 13 days prior on doubling rate. The black curves are the concurrent effect functions and the orange ribbons represent 95% pointwise confidence bands. The variable $W(t-13)$ represents the percent change in workplace mobility lagged by 13 days and is generally but not always negative.

We adopt the functional concurrent regression (FCR) models,

1 $$\begin{aligned} \gamma (t)&=\beta _0(t) + \beta _1(t) P + \beta _2(t)A + \beta _3(t) C(t) + \beta _4(t)W(t-\Delta ) + Z(t) \end{aligned}$$

2 $$\begin{aligned} \frac{d}{dt}\text {CFR}(t)&= \beta _0(t) + \beta _1(t) P + \beta _2(t)A + \beta _3(t)\text {CFR}(t) + Z(t), \end{aligned}$$

where the predictors consist of population density *P*, the proportion of the population over age 65 *A*, the log-cumulative case counts per million *C*(*t*), and the lagged decrease in workplace mobility $W(t-\Delta )$. The former two predictors are baseline covariates while the latter two are time-varying. The error term *Z*(*t*) denotes a mean-zero stochastic drift process. The optimal lag $\Delta$ is chosen data-adaptively and was found to maximize predictive power at $\Delta =13$ days (“Methods”: “Empirical dynamics”). Concurrent effect functions for each regressor are displayed alongside their 95% confidence bands in Fig. 7. A stretch of time where the confidence band does not touch 0 suggests that the effect of that predictor is locally significant during that time interval.

##### Doubling rates

Higher doubling rates reflect faster spread of infection and our analysis does not provide evidence that they are associated with higher population density. The fraction of population over age 65 has a significant but complex effect on doubling rates. During the priming period, the doubling rate is positively correlated with demographically older populations, but this effect goes into reverse from day 25 on, perhaps as the presence of older members of society promotes additional self-isolation; however it is quite possible that this association is due to the potential confounding effect that many later mobility-reducing countries have older populations.

The additional effect of total cases per capita is significantly positive at the very start, but the concurrent regression slope estimate tends to be negative in the following period, which portends a dynamic regression to the mean effect^28^, i.e., countries with doubling rates away from the mean tend to gravitate back to the mean during the early time period, reflecting declining variation across countries. The mean curve in Fig. 6 indicates monotone declining doubling rates, so countries which did not have many cases in this period tend to catch up to the mean behaviour. Doubling rates are for the most part positively correlated with change in workplace mobility after a lag of 13 days, which suggests that reduced mobility, which corresponds to a negative change, may significantly slow the growth rate of confirmed cases, though this benefit is only seen approximately 2 weeks later.

**Figure 8:**
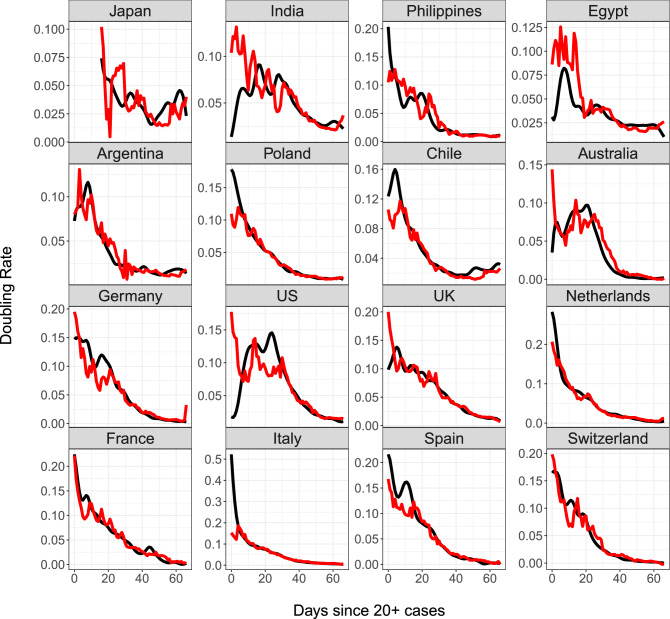
Observed (black) and predicted (red) trajectories for doubling rates of COVID-19 in sixteen countries, where predictors are total cases on log scale and change in workplace mobility with a 13 day lag. Change in workplace mobility is as presented in Fig. 5.

Figure 8 shows the predicted doubling rate for sixteen countries according to the historical functional linear model (see Section S.4 in the Supplement). In the case of Japan, predictions are only possible after day 15 as mobility information prior to this date is not available. Periods where the observed doubling rate is higher than predicted reflect worse performance than expected based on history and mobility. Predictions of doubling rates are poor in the initial priming period for most countries, but improve later on, which may be a consequence of the dynamic regression to the mean effect as visualized in Fig. 6a. Countries with difficult to predict trajectories include Japan, India, Egypt, and the US. This may be due to low testing rates, as these countries initially report much fewer tests administered in comparison to others. The US reported an exceedingly low doubling rate in first week, potentially reflecting low levels of testing. Observed doubling rates were in line with predictions only after day 35, likely after more testing efforts were implemented. Poland also performed worse than expected at first, but its doubling rate dropped to the predicted rate at day 10. Most countries saw improvements in the doubling rate about 1.5–2 weeks after workplace mobility began to drop. This corroborates the idea that lockdown policies are effective at reducing spread after a short period of delay.

##### Case fatality rates

**Figure 9:**
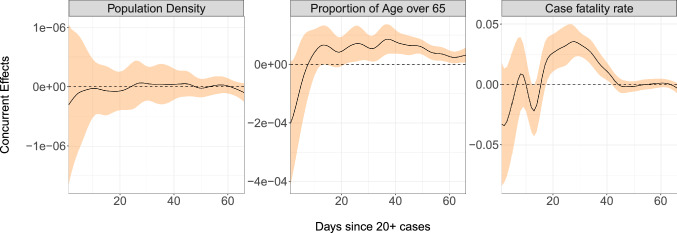
Time-varying regression effects of population density, percentage of population over age 65, and case fatality rates on the derivative of case fatality rate. The black curves are the regression slopes and the orange ribbons represent 95% pointwise confidence bands.

Case fatality rates exhibit dynamic explosive behavior as seen in the effect curve of CFR(*t*) in the third panel of Fig. 9, which is positive after day 15. This means that case fatality rates either above or below the mean fatality rate curve in Fig. 6 tend to move even further away from the mean as time progresses, as seen in Italy’s curve, for example. A possible explanation for this could be that an already overwhelmed healthcare system is predestined for future worse outcomes as the disease continues to spread and resources become more and more scarce. Higher case fatality rates generally do not correlate strongly with population density, but are positively associated with demographically older countries, which is not surprising.

## Discussion

In this study, we have explored a battery of time-varying approaches for modeling the cumulative COVID-19 case trajectories by pooling data across countries, which is facilitated by the curve-based methodology of functional data analysis. These techniques provide a natural and intuitive framework for comparing case trajectories, constructing principled forecasts for future case counts, and quantifying the time-varying effects of covariates. A major strength of the functional data approach is the borrowing of information across countries, i.e., models and predictions for case counts carry knowledge gained from the entire sample of countries and not just that of a single country’s data. This approach enables the modeling of time-varying associations and results in dynamic effects which are reflective of the changing nature of the infectivity cycles and their dependence on covariates. It also offers a flexible and non-parametric alternative to the more rigidly-structured and less data-oriented epidemiological models.

The advantages afforded by this functional approach are not immune to limitations imposed by data quality, however. The bias in COVID-19 case counts is difficult to control for, as there are manifold causes of under-reporting (e.g., the delay between infection and confirmation, confounding through testing availability, prevalence of asymptomatic carriers, etc.). Findings regarding individual countries must be evaluated in light of the their unique set of biases. Indeed, this issue does not have a clear solution and warrants continued attention. A feature of our analysis that is favorable is that while absolute case counts may be subject to reporting error, the trends in time-dynamics on which we focus here are likely more robust, in the sense that biases for a given country may affect the entire trajectory, although to a different degree over time as for example testing is ramped up.

Applying functional PCA led to the discovery of four main patterns of disease progression since initial exposure and to identify the countries that represent these archetypes. A complementary analysis of percentlle rank dynamics allows for comparison of relative performance of countries at different points in time. In terms of predictive modeling, we introduce a dynamic FPCA approach for forecasting country-specific case counts, illustrated with 10-day forecasts. These forecasts, when compared to the eventually observed curves, can illustrate whether a country over- or under-performs during a given shorter time period. Lastly, we apply concurrent regression to quantify the effects of baseline and time-varying covariates, such as population density and reduction in social mobility over time. The results of our analysis can be summarized in the following seven key messages, where we also highlight the underlying methodology:

- The mean trajectory of log-cumulative case counts per capita increases linearly (representing exponential growth) until around day 25 at which time it starts to flatten. Variations from this mean trend follow one of two patterns. The first pattern corresponds to a baseline level of viral spread which changes little over time; the second corresponds to a marked increase or decrease of the transmission rate after the first 25 days of exposure (Functional PCA, Figs. 2 and 3).
- A country’s relative performance in terms of prevention stabilizes after an approximately month-long priming period. How well a country handled the situation during this initial window largely determined their final standing after 67 days of exposure (Rank Dynamics, Fig. 4).
- Countries which were late to reduce social activity perform worse both relative to other countries and in terms of their own projected case counts (Dynamic FPCA, Fig. 5).
- Trajectories of countries with earlier exposure times contain valuable information for forecasting case counts for more recently exposed nations. The curve-based methods of functional data analysis are uniquely suited for pooling information across a sample of trajectories, including those which are only partially observed. (Dynamic FPCA, Fig. 5)
- Reduction in workplace mobility is associated with lower spread of the virus, though the effects are lagged. Benefits of reduced social mobility are delayed by roughly 2 weeks (Functional Concurrent Regression).
- Baseline demographics are significantly associated with doubling rates during specific time windows. There was no evidence for a systematic effect of population density, while demographically older countries typically experienced higher rates of spread during the first 25 days of exposure before course-correcting and enjoying lower doubling rates thereafter (FCR, Figs. 6, 7 and 8); the latter may be a confounding effect.
- Case fatality rates exhibit positive-feedback patterns of severity. After the first 2 weeks of exposure, higher than average case fatality rates foreshadow increased mortality in the future. Meanwhile, countries which have lower than average case fatality rates early on typically improve upon these rates even further down the line. Demographically older countries suffer higher case fatality rates, as expected. (FCR, Figs. 6 and 9)

The first three key messages highlight the importance of an immediate response to rising caseloads, particularly during the first 25 days, a period that we have reason to characterize as a priming period. For example, the UK and Greece both have average baseline levels of spread, but Greece successfully maintains a level of containment while the UK suffers increased spread of the virus. The differences in initial responses across countries with similar baselines may be key to understanding the factors that cause a country’s case load to escalate rapidly. Previous studies (see, e.g.^31^) have identified the first few weeks as a critical time period for management of the virus, with some European countries generally being less prepared to contain spread. These findings are reinforced by our functional principal component analysis, in which the majority of European countries exhibit either high baseline levels of spread or a dramatic increase in transmission rate after the first 25 days.

The positive-feedback behavior seen in case fatality rates may reflect variation in the ability of healthcare systems to cope with surges of cases, but could also be due to improvements in attribution of cause of death over time. Khafaie and Rahim^38^ found that early access to medical care plays a key role in decreasing fatality rates and suggested that under-supplied countries may not have the resources to efficiently implement intervention, affecting patients in high-risk groups negatively. Conversely, when case fatality rates are low, the positive feedback loop trending away from the mean that we find in the data is driving fatality rates even lower.

The importance of enacting preventative measures early, which has been previously illustrated in, for example^7^, and^10^, is reaffirmed by our results. We found that countries where decreased social mobility was enacted before reaching higher levels of spread did better than their predicted case counts and generally had less severe situations at the end of the time period that we considered. We observed a 2 week delay of the effect of decreased mobility on caseload, which is in line with existing lag estimates, generally characterized as between 9 and 14 days^7,9,35^. Generally, our analysis reveals that the impact of factors that are predictive for caseload or case fatality rates varies over time. This indicates that prevention measures will be most effective when they are closely tied to the dynamics of the pandemic.

The code for the functional data analysis methods used in this study is publicly available in the R package fdapace^39^.

## Methods

### Data

Our analysis focuses on modeling and predicting the cumulative number of cases per million individuals in log scale, using information obtained from the COVID-19 Data Repository by the Center for Systems Science and Engineering (CSSE) at Johns Hopkins University, which was accessed on May 18, 2020. The data consist of the cumulative number of confirmed cases and deaths per day since Jan 22, 2020 for several countries and is publicly available on Github at https://github.com/CSSEGISandData/COVID-19. Since the pandemic reached individual countries at staggered times, we consider a time interval $\mathcal {T} = [0,66]$ consisting of 67 days where the initial time $t=0$ represents the earliest date at which at least 20 confirmed cases were reported.

The cumulative number of cases per million individuals on the log scale is formally defined as

$$\begin{aligned} C(t)=\log _{10} \left( \frac{\# \text {confirmed cases up to day } t }{ \text {Population size }} \times 10^6 \right) , \end{aligned}$$

where $t\in [0,66]$. Defining the initial time $t=0$ in such a way aligns trajectories according to the onset of the pandemic in a given country and allows for temporal comparisons across nations even as they were first exposed to the virus at different times. We include in our analysis countries with population size at least 100,000 by 2018 that have suffered at least 5 deaths by May 18, 2020 and have been exposed to the virus for at least 67 days. This resulted in $n=64$ countries that were included in the analysis. A table of the selected countries and of the dates corresponding to $t=0$ for all countries can be found in Supplement S.1.

Data for population density and the demographic fraction of the population 65 years old or older as of 2018 was obtained from the World Bank database, available at https://www.worldbank.org/. For Iran we used the 2017 data as the data for 2018 were unavailable. For Taiwan, covariate information was not available at the World Bank database and was obtained from https://www.indexmundi.com/taiwan/#Demographics. We also use Google community mobility data as time-varying covariates, available at https://www.google.com/covid19/mobility/. The following countries did not have Google mobility data available: Albania, Algeria, China, Iceland, Iran, and Russia. See Supplement S.1 for more details.

### Functional principal component analysis

Functional principal component analysis (FPCA) is derived from a functional analogue of the spectral decomposition for covariance matrices. Considering a generic square-integrable stochastic process $C(t),\ t \in \mathcal {T}$, with mean function $\mu (t) = {E}(C(t))$ and covariance function $G(s, t) = \text {Cov}(C(s), C(t))$, $s,t \in \mathcal {T}$, under mild regularity conditions *G*(*s*, *t*) admits an orthogonal expansion

3 $$\begin{aligned} G(s, t) = \sum _{k=1}^\infty \lambda _k \varphi _k(s) \varphi _k(t), \end{aligned}$$

where $\lambda _1 \ge \lambda _2 \ge \dots > 0$, $\sum _{k=1}^\infty \lambda _k <\infty$, are the eigenvalues and $\varphi _1$, $\varphi _2 \dots$ are the (orthonormal) eigenfunctions of the Hilbert-Schmidt autocovariance operator $A_G: f\in L^2(\mathcal {T}) \mapsto \int _\mathcal {T} G(s, t) f(s) ds$.

With this decomposition, the Karhunen–Loève representation theorem states that

4 $$\begin{aligned} C(t) = \mu (t)+\sum _{k=1}^\infty \xi _k \varphi _k(t), \end{aligned}$$

where the scores $\xi _k = \int _\mathcal {T} (C(t) - \mu (t)) \varphi _k(t) dt$ satisfy $E(\xi _k) = 0, \text {Var}(\xi _k) = \lambda _k \text { and } E(\xi _k \xi _l) = 0 \text { for } k \ne l$. Here $\xi _k$ is the functional principal component score (FPC) of $X(\cdot )$ associated with the $k^{\text {th}}$ eigenfunction $\varphi _k$. Thus, the FPC scores are projections of the centered stochastic process onto the directions given by the eigenfunctions and summarize how a function changes from the mean curve along the principal modes of variation. Moreover, from (4) the centered process is equivalent to $(\xi _1, \xi _2, \dots )^T$.

By truncating the representation in (4) to a finite number of *K* components one achieves dimension reduction and can approximate the original stochastic process through its most important modes of variations. Plugging in estimates for mean function $\hat{\mu }$, eigenfunctions $\hat{\phi }_k$ and FPC scores $\hat{\xi }_k,$ FPCA then provides finitely truncated fits for the random trajectories $C(\cdot )$,

5 $$\begin{aligned} \hat{C}(t) = \hat{\mu }(t)+\sum _{k=1}^K \hat{\xi }_k \hat{\varphi }_k(t). \end{aligned}$$

Here *K* is often chosen so that the fraction of variability explained,

$$\text {FVE}(K)=\sum _{k=1}^K \hat{\lambda }_k /\sum _{k=1}^\infty \hat{\lambda }_k,$$

is above a threshold, e.g. 97%. For the COVID-19 cases, the choice $K=2$ meets this criterion. Further details can be found in^24,25,33^.

The modes of variation illustrate the individual effect that each of the scores has on the function *C*(*t*). The first mode of variation corresponds to the curve $\mu (t)+\alpha \sqrt{\lambda _1} \phi _1(t)$, where $\alpha$ ranges in the interval $[-2,2]$. This represents the effect of the first score on *C*(*t*) as it varies between $\pm 2$ standard deviations away from the mean. Similarly, the second mode of variation is obtained by replacing $\lambda _1$ and $\phi _1(t)$ by $\lambda _2$ and $\phi _2(t)$, respectively, in the previous expression, where in implementations these population quantities are replaced by estimates.

### Dynamic functional principal components

The approximation in (5) provides a straightforward method for predicting a curve $C_i(t)$, if we have access to estimates of the mean function, eigenfunctions, and the FPC scores. We follow the Principal Components Analysis through Conditional Expectation (PACE) approach proposed for sparse longitudinal data^33^. For dynamic forecasting we proceed as follows.

Suppose there are *m* countries for which trajectories have been observed for $t_0+ \Delta t$ days, forming a training sample. That is, for the $i^{th}$ country in the training sample, $i = 1 ,\ldots , m$, the current span of observations $[0,T_i]$ is such that $T_i> t_0+ \Delta t$. If the number of countries *m* in the training sample is sufficiently large, it is promising to predict the future trajectory of a $m+1^{st}$ country that is not part of the training sample by borrowing information from the countries in the training sample for which trajectories have already been observed for the time span to be predicted. Suppose we have data for the $m+1^{st}$ country until time $t_0$ and are interested in predicting its trajectory for the next $\Delta t$ days, i.e., prediction of the trajectory in the time interval $[t_0,t_0+ \Delta t]$, where $\Delta t$ is chosen to be reasonably small, based on the observed data vector $C_{m+1}= (C_{m+1}(0),\dots ,C_{m+1}(t_0))^T$.

One can then recover the process $\vec {C}_{m+1}(t)$ on $[0,t_0+ \Delta t]$ by using $\{C_{i}(t_{ij})\}$, where $i= 1,\ldots ,m$ and $0 \le t_{ij} \le t_0+ \Delta t$. That is to say the forecast is obtained by using observations between day 0 to $t_0+ \Delta t$ from the countries in the training sample, for which trajectories $\{C_{i}(t_{ij})\}$ have been observed for the time period $[0,t_0+ \Delta t]$. Then the estimated Karhunen–Loève representation (5) of $C_{m+1}(t)$ on the interval $[0,t_0+ \Delta t]$ is

6 $$\begin{aligned} \hat{C}_{m+1}(t)=\hat{\mu }(t) + \sum _{k=1}^{K}\hat{\xi }_{n+1,k} \hat{\varphi }_k(t),\quad t\in [0,t_0+\Delta t], \end{aligned}$$

where $\hat{\mu }(t)$ is estimated either cross-sectionally or with a smoothing method, $\hat{\varphi }_k$ and $\hat{\lambda }_k$ are estimated using the truncated spectral decomposition of the covariance surface, and the scores are estimated using the PACE approach^33^, where the $k^{th}$ score for trajectory $(m+1)$ is

7 $$\begin{aligned} \hat{\xi }_{m+1,k}=\hat{\lambda }_k \hat{\varphi }_k^T \hat{\Sigma }_{t_0}^{-1} (\vec {C}_{m+1}-\hat{\mu }), \end{aligned}$$

where $\vec {C}_{n+1}= (C_{m+1}(0),\dots ,C_{m+1}(t_0))^T$ is the observed vector of data for the trajectory to be predicted, $\hat{\mu }$ is estimated mean vector which equals to $\hat{\mu }(t)$ evaluated at $t=(0,\dots ,t_0)^T$, and $\hat{\Sigma }_{t_0}^{-1}$ is the $(t_0+1) \times (t_0+1)$ estimated variance-covariance matrix for $\vec {C}= (C(0),\dots ,C(t_0))^T$ based on the entire sample.

We then iterate this process for each country of interest, and update our prediction moving forward. For forecasting COVID-19 cases, we display results for predicting $\Delta t=10$ days at a time.

### Empirical dynamics

The severity of infectious disease spread is commonly modeled using doubling time and case fatality rates. Here we describe time-varying regression models for these quantities inspired by empirical dynamics^28^, aiming to systematically model derivatives of smooth processes using the process itself as predictor, which can be characterized as dynamics learning. Additional technical details about empirical dynamics can be found in Section S.5 of the Supplement.

#### Doubling rates

Let $N_c(t)$ denote the total confirmed cases per million at time *t*. Then the doubling time $\kappa (t)$ at time *t* is the length of time necessary for the cumulative cases per million to double. In other words, $\kappa (t)$ is defined explicitly by the relation

8 $$\begin{aligned} \frac{N_c(t+\kappa (t))}{N_c(t)}=2. \end{aligned}$$

By a first order Taylor series expansion, a linear approximation of the numerator in (8) is

9 $$\begin{aligned} N_c(t+\kappa (t)) \approx N_c(t) + \frac{d}{dt}N_c(t) \kappa (t). \end{aligned}$$

The approximation in (9) together with the fact that $N_c(t+\kappa (t))=2N_c(t)$ leads to

$$\begin{aligned} \kappa (t)=\frac{N_c(t)}{\frac{d}{dt}N_c(t)}=\frac{1}{\frac{d}{dt} C(t)}, \end{aligned}$$

where the representation in terms of *C*(*t*) follows from the substitution $C(t)=\log _{10}\left( N_c(t) \right)$.

The *doubling rate* $\gamma (t)$ is defined as

$$\begin{aligned} \gamma (t)=\frac{1}{\kappa (t)}=\frac{d}{dt} C(t). \end{aligned}$$

The doubling rate quantifies the rate of spread, with lower doubling rates corresponding to longer doubling times. This naturally leads us to model the empirical dynamics of the process *C*(*t*) by

10 $$\begin{aligned} \gamma (t)=\frac{d}{dt}C(t)= \beta _0(t)+ \beta _1(t) C(t)+ \beta _2(t) P+ \beta _3(t) A + \beta _4(t) W(t-\Delta )+ Z(t) \end{aligned}$$

for $t>\Delta$ where *P* represents population density, *A* represents the fraction of the population over age 65, $W(t-\Delta )$ denotes the percentage change from baseline of workplace mobility patterns at time $t-\Delta$, and *Z*(*t*) denotes a mean zero stochastic drift process. We use the data adaptive criterion described in Section S.5 of the Supplement to select an optimal choice of lag $\Delta$, which for our analysis is 14 days. Figures 3 and 4 in Section S.7 of the Supplement illustrate the strong multi-collinearity among the different community mobility patterns, so we utilize only workplace mobility. Note that in (10) we model the doubling rate $\gamma (t)$ instead of the doubling time $\kappa (t)$, which circumvents the numerical issues involved with a vanishing $\frac{d}{dt}C(t)$. For obtaining the observed doubling rate trajectories, we estimate the derivative of *C*(*t*) using local quadratic smoothing^40,41^ with a bandwidth of 2 days.

For doubling rate prediction, we consider the functional linear regression model^42,43^ that uses the entire history from $t-13$ to $t-1$ days,

11 $$\begin{aligned} \frac{d}{dt}C(t)= \beta _0(t)+ \int _{t-13}^{t-1}\beta _1(t,s) C(s)ds+ \int _{t-13}^{t-1}\beta _2(t,s) W(s)ds + Z(t). \end{aligned}$$

Further details can be found in Section S.5 of the Supplement.

#### Case fatality rates

The case fatality rate at time *t* is the ratio of the total death count and the total case count at that time. Letting $N_d(t)$ denote the total death count per million at time *t*, the case fatality rate is $$\begin{aligned} \text {CFR}(t)=\frac{N_d(t)}{N_c(t)}. \end{aligned}$$ For the dynamics of fatality rates, we consider the concurrent model

12 $$\begin{aligned} \frac{d}{dt}\text {CFR}(t)= \beta _0(t)+ \beta _1(t) \text {CFR}(t)+ \beta _2(t) P+ \beta _3(t) A + Z(t), \end{aligned}$$

where *P*, *A*, and *Z* are defined as in the previous section.

## Supplementary information

Supplementary Information.

## Data Availability

Data that support the findings of this study are publicly available from the following sources: Johns Hopkins University CSSE (https://github.com/CSSEGISandData/COVID-19), World Bank (https://www.worldbank.org/), Indux Mundi (https://www.indexmundi.com/taiwan/#Demographics), and Google (https://www.google.com/covid19/mobility/). Scripts are publicly available in the R package fdapace (https://cran.r-project.org/web/packages/fdapace/index.html).

## References

[1] Johns Hopkins University. Global cases by the center for systems science and engineering (CSSE) at Johns Hopkins University. Coronavirus Resource Center (2020).

[2] Hoffmann M (2020). SARS-CoV-2 cell entry depends on ACE2 and TMPRSS2 and is blocked by a clinically proven protease inhibitor. Cell.

[3] Anastassopoulou C, Russo L, Tsakris A, Siettos C (2020). Data-based analysis, modelling and forecasting of the COVID-19 outbreak. PLoS One.

[4] Peng, L., Yang, W., Zhang, D., Zhuge, C. & Hong, L. Epidemic analysis of COVID-19 in China by dynamical modeling. arXiv:2002.06563 (2020).

[5] Sun G-Q, Xie J-H, Huang S-H, Jin Z, Li M-T, Liu L (2017). Transmission dynamics of cholera: mathematical modeling and control strategies. Commun. Nonlinear Sci. Numer. Simul..

[6] Petropoulos F, Makridakis S (2020). Forecasting the novel coronavirus COVID-19. PLoS One.

[7] Wagner AB (2020). Social distancing merely stabilized COVID-19 in the United States. Stat.

[8] Lau, H. *et al*. The positive impact of lockdown in Wuhan on containing the COVID-19 outbreak in China. *J. Travel Med.* 2020. 10.1093/jtm/taaa037 PMC7184469 32181488

[9] Tian H (2020). An investigation of transmission control measures during the first 50 days of the COVID-19 epidemic in China. Science.

[10] Li M-T (2020). Analysis of COVID-19 transmission in Shanxi Province with discrete time imported cases. Math. Biosci. Eng..

[11] Pellis, L. *et al.* Challenges in control of COVID-19: short doubling time and long delay to effect of interventions. arXiv:2004.00117 (2020). 10.1098/rstb.2020.0264 PMC8165602 34053267

[12] Zhanwei D (2020). Risk for transportation of coronavirus disease from Wuhan to other cities in China. Emerg. Infect. Dis..

[13] Zhang, I. & Lin, G. Spatiotemporal analysis for the outbreak of COVID-19 in the world. Available at SSRN 3576816 (2020).

[14] Kucharski AJ (2020). Early dynamics of transmission and control of COVID-19: a mathematical modelling study. Lancet Infect. Dis..

[15] Lee, P. H. Estimating the real-time case fatality rate of COVID-19 using Poisson mixtures model. medRxiv (2020).

[16] Oliveiros, B., Caramelo, L., Ferreira, N. C. & Caramelo, F. Role of temperature and humidity in the modulation of the doubling time of COVID-19 cases. medRxiv (2020).

[17] Caramelo F, Ferreira N, Oliveiros B (2020). Estimation of risk factors for COVID-19 mortality-preliminary results. medRxiv.

[18] Gao, S., Rao, J., Kang, Y., Liang, Y. & Kruse, J. Mapping county-level mobility pattern changes in the united states in response to COVID-19. Available at SSRN 3570145 (2020).

[19] Engle, S., Stromme, J. & Zhou, A. Staying at home: mobility effects of COVID-19. Available at SSRN (2020)

[20] Park M, Cook AR, Lim JT, Sun Y, Dickens BL (2020). A systematic review of COVID-19 epidemiology based on current evidence. J. Clin. Med..

[21] Lachmann, A. Correcting under-reported COVID-19 case numbers. medRxiv (2020).

[22] Krantz, S. G. & Srinivasa Rao, A. S. R. Level of under-reporting including under-diagnosis before the first peak of COVID-19 in various countries: Preliminary retrospective results based on wavelets and deterministic modeling. *Infect. Control Hosp. Epidemiol.*, 1–8 (2020). 10.1017/ice.2020.116 PMC7170968 32268929

[23] Ramsay, J. O. & Silverman, B. W. *Functional data analysis. Springer Series in Statistics* (2nd ed.). Springer, New York (2005).

[24] Horvath L, Kokoszka P (2012). Inference for Functional Data with Applications.

[25] Wang J-L, Chiou J-M, Müller H-G (2016). Functional data analysis. Annu. Rev. Stat. Appl..

[26] Jiang C-R, Wang J-L (2010). Covariate adjusted functional principal component analysis. Ann. Stat..

[27] Lin Z, Wang L, Cao J (2016). Interpretable functional principal component analysis. Biometrics.

[28] Müller H-G, Yao F (2010). Empirical dynamics for longitudinal data. Ann. Stat..

[29] Castro PE, Lawton WH, Sylvestre EA (1986). Principal modes of variation for processes with continuous sample curves. Technometrics.

[30] Han K (2018). Functional component analysis for identifying multivariate patterns and archetypes of growth, and their association with long-term cognitive development. PLoS One.

[31] Khosrawipour V (2020). Failure in initial stage containment of global COVID-19 epicenters. J. Med. Virol..

[32] Chen Y, Dawson M, Müller H-G (2020). Rank dynamics for functional data. Comput. Stat. Data Anal..

[33] Yao F, Müller H-G, Wang J-L (2005). Functional data analysis for sparse longitudinal data. J. Am. Stat. Assoc..

[34] Google LLC. Google COVID-19 community mobility reports. https://www.google.com/covid19/mobility. Last accessed May 18, 2020.

[35] Badr, H., Du, H., Marshall, M., Dong, E., Squire, M. & Gardner, L. M. Social distancing is effective at mitigating COVID-19 transmission in the United States. medRxiv (2020).

[36] Promislow D (2020). A geroscience perspective on COVID-19 mortality. J. Gerontol. Ser. A.

[37] Cauchemez S (2014). Middle east respiratory syndrome coronavirus: quantification of the extent of the epidemic, surveillance biases, and transmissibility. Lancet Infect. Dis..

[38] Khafaie MA, Rahim F (2020). Cross-country comparison of case fatality rates of COVID-19/SARS-COV-2. Osong Public Health Res. Perspect..

[39] Carroll , C. *et al.**fdapace: Functional Data Analysis and Empirical Dynamics*. R package version 0.5.5 (2020)

[40] Müller H-G, Stadtmüller U, Schmitt T (1987). Bandwidth choice and confidence intervals for derivatives of noisy data. Biometrika.

[41] Fan J, Gijbels I (1996). Local Polynomial Modelling and its Applications: Monographs on Statistics and Applied Probability 66.

[42] Cardot H, Ferraty F, Sarda P (1999). Functional linear model. Stat. Probab. Lett..

[43] Şentürk D, Müller HG (2010). Functional varying coefficient models for longitudinal data. J. Am. Stat. Assoc..

