# Supplementary material for: Time dynamics of COVID-19
Source: Sci Rep. 2020 Dec 3;10:21040. doi: 10.1038/s41598-020-77709-4 (PMC7712909; doi:10.1038/s41598-020-77709-4)
Supplement: Supplementary file 1 — Supplementary Information. [file 41598_2020_77709_MOESM1_ESM.pdf]

Time Dynamics of COVID-19: Supplementary Material

Cody Carroll[a], Satarupa Bhattacharjee[a,1], Yaqing Chen[a,1],

Paromita Dubey[b,1], Jianing Fan[a,1], Álvaro Gajardo[a,1],

Xiner Zhou[a,1], Hans-Georg Müller[a,2*], Jane-Ling Wang[a,3]

[a] Department of Statistics, University of California, Davis

Davis, CA 95616 USA

[b] Department of Statistics, Stanford University

Stanford, CA 94305 USA

May 2020

* Corresponding author: hgmueller@ucdavis.edu

[1] These authors contributed equally to this work.

[2] Research supported by NSF DMS-1712862

[3] Research supported by NSF DMS-1914917

KEY WORDS: COVID-19, Cases and Deaths, Doubling Rates, Case Fatality Rates, Functional Data Analysis, Functional Principal Component Analysis, Empirical Dynamics, Concurrent Regression.

S.1 Details on data pre-processing

Table 1 presents information such as the first up-crossing time of 20 confirmed cases for each country in the study. The cross-sectional ranks and integrated ranks are estimated as per Section S.6.

The Google community mobility trends data measure how the frequency and length of stay at different locations have changed relative to a baseline level prior to the COVID-19 pandemic. The baseline activity level is defined as the median activity value for the corresponding day of the week in the period of Jan. 3 to Feb. 6, 2020 during which time most countries (excluding China) did not implement any distancing efforts. Available categories include retail, grocery, park, transit, workplace, and residential categories. Since these categories are highly collinear we consider just workplace mobility as an index of social activity (see Section S.7).

Country	First up-crossing date	Final observation date	Rank at $t = 5$	Rank at $t = 65$	Integrated rank
Luxembourg	Mar 13	May 18	0.98	0.97	0.98
Iceland	Mar 04	May 09	0.95	0.95	0.97
Ireland	Mar 09	May 14	0.84	0.94	0.90
Switzerland	Mar 01	May 06	0.83	0.89	0.89
Qatar	Mar 10	May 15	0.94	0.98	0.89
Belgium	Mar 04	May 09	0.81	0.91	0.87
Spain	Feb 28	May 04	0.45	0.92	0.83
Portugal	Mar 07	May 12	0.48	0.80	0.81
Estonia	Mar 13	May 18	0.97	0.53	0.79
Austria	Mar 03	May 08	0.64	0.64	0.79
Italy	Feb 21	Apr 27	0.53	0.88	0.78
Norway	Mar 02	May 07	0.86	0.56	0.78
Panama	Mar 13	May 18	0.80	0.73	0.78
Netherlands	Mar 03	May 08	0.72	0.77	0.78
Denmark	Mar 06	May 11	0.91	0.66	0.76
Sweden	Mar 03	May 08	0.78	0.75	0.73
Israel	Mar 05	May 10	0.59	0.67	0.71
Slovenia	Mar 10	May 15	0.92	0.42	0.68
France	Feb 27	May 03	0.39	0.78	0.67
Serbia	Mar 13	May 18	0.66	0.58	0.67
Finland	Mar 08	May 13	0.88	0.50	0.64
Bahrain	Feb 25	May 01	0.89	0.69	0.63

Germany	Feb 26	May 02	0.30	0.70	0.62
Czech Rep.	Mar 08	May 13	0.69	0.44	0.61
Croatia	Mar 13	May 18	0.77	0.41	0.61
Chile	Mar 11	May 16	0.56	0.72	0.59
Belarus	Mar 13	May 18	0.47	0.84	0.57
UK	Feb 28	May 04	0.25	0.81	0.56
Peru	Mar 13	May 18	0.44	0.83	0.56
Iran	Feb 22	Apr 28	0.36	0.52	0.55
Romania	Mar 10	May 15	0.50	0.45	0.51
Albania	Mar 12	May 17	0.73	0.34	0.49
US	Feb 24	Apr 30	0.03	0.86	0.48
Slovakia	Mar 13	May 18	0.75	0.31	0.47
Canada	Feb 29	May 05	0.19	0.61	0.44
Greece	Mar 05	May 10	0.58	0.27	0.43
Saudi Arabia	Mar 10	May 15	0.41	0.55	0.42
Bulgaria	Mar 13	May 18	0.67	0.33	0.42
Poland	Mar 10	May 15	0.42	0.39	0.42
Kuwait	Feb 26	May 02	0.70	0.48	0.41
UAE	Feb 29	May 05	0.38	0.59	0.40
Costa Rica	Mar 12	May 17	0.55	0.17	0.39
Australia	Feb 29	May 05	0.34	0.30	0.37
Russia	Mar 10	May 15	0.05	0.62	0.34
Georgia	Mar 11	May 16	0.61	0.20	0.34
Lebanon	Mar 06	May 11	0.62	0.14	0.33
Brazil	Mar 08	May 13	0.12	0.47	0.29
S. Korea	Feb 06	Apr 12	0.09	0.25	0.26
Singapore	Feb 04	Apr 10	0.52	0.36	0.26
S. Africa	Mar 13	May 18	0.31	0.28	0.25
Argentina	Mar 13	May 18	0.28	0.22	0.25
Mexico	Mar 13	May 18	0.17	0.38	0.24
China	Jan 22	Mar 28	0.33	0.06	0.22
Algeria	Mar 09	May 14	0.16	0.16	0.17
Pakistan	Mar 12	May 17	0.23	0.23	0.16
Philippines	Mar 09	May 14	0.20	0.12	0.15
Malaysia	Feb 15	Apr 21	0.11	0.19	0.15
Iraq	Mar 02	May 07	0.27	0.09	0.12
Egypt	Mar 08	May 13	0.14	0.11	0.11
Indonesia	Mar 10	May 15	0.06	0.08	0.07
Taiwan	Feb 16	Apr 22	0.22	0.00	0.07
Thailand	Feb 04	Apr 10	0.08	0.03	0.03
India	Mar 04	May 09	0.00	0.05	0.02
Japan	Feb 01	Apr 07	0.02	0.02	0.02

Table 1: First up-crossing times during 2020 of 20+ confirmed cases and dates after the 67 days time window for each of the 64 countries in the study along with their cross-sectional ranks at $t = 5$ and $t = 65$ days, as well as the integrated rank.

S.2 Fitted Trajectories

Figure 1 shows the imputed curves for the confirmed cases through the FPCA method with $K = 2$ eigenfunctions for the countries Brazil, Chile, India, Italy, Switzerland, United Kingdom, and the United States. The first 2 eigenfunctions explain 97% of the total variation and the quality of the fits suggests that including just two components in the Karhunen-Loève expansion works well.

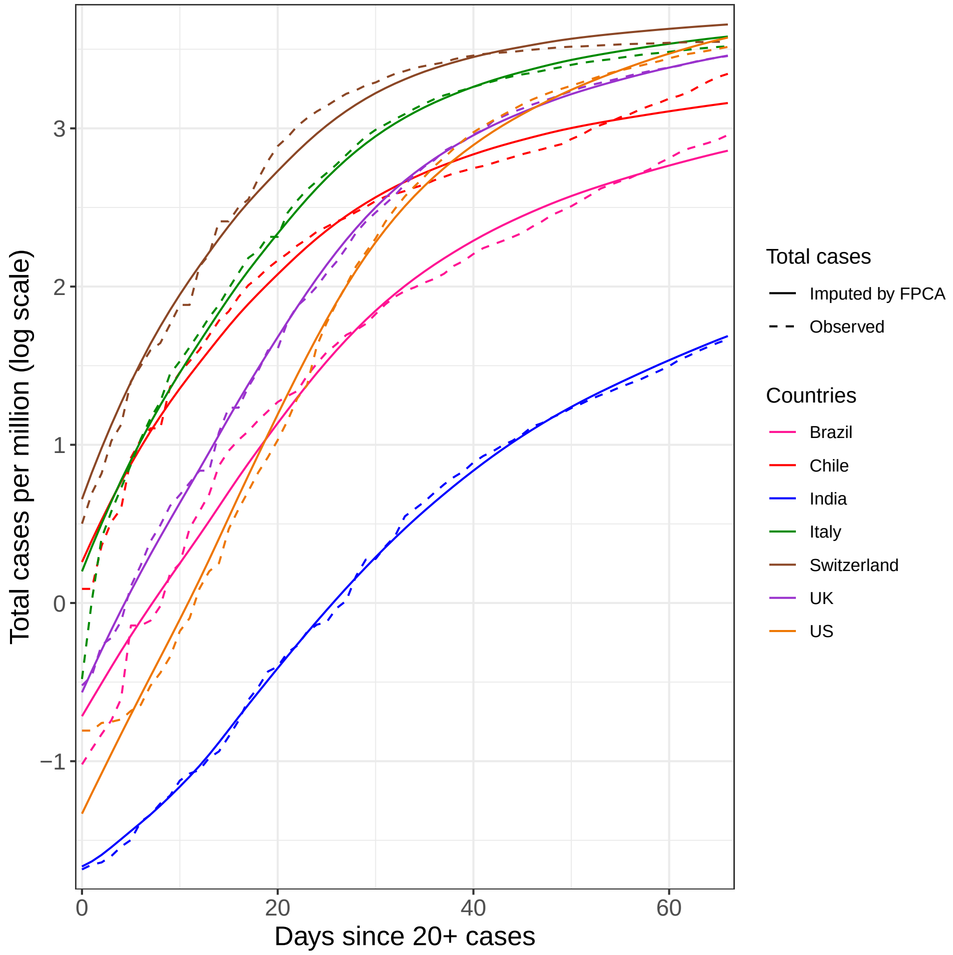

Figure 1: Observed (dashed) and estimated (solid) caseload curves $C(t)$, for selected countries.

S.3 Functional Concurrent Regression

For an observed response curve $Y(t)$, functional predictor $X(t)$ and baseline covariate U, the functional concurrent regression model is given by

$$Y(t) = \beta_0(t) + \beta_1(t)X(t) + \beta_2(t)U + \epsilon(t),$$

where $\beta_0(t)$, $\beta_1(t)$ and $\beta_2(t)$ are smooth coefficient functions and $\epsilon(t)$ is a zero mean Gaussian process. Various estimation techniques are available for the intercept function $\beta_0(t)$ and the slope functions $\beta_1(t)$ and $\beta_2(t)$ for both densely and sparsely observed functional data [1, 2, 3].

For assessing the goodness of fit, one can use the dynamic coefficient of determination

$$R^2(t) = 1 - \frac{\mathrm{var}(\epsilon(t))}{\mathrm{var}\,(Y(t))}.$$

Larger values of $R^2(t)$ indicate that a larger fraction of the variability in the response $Y(t)$ is explained by the linear model in $X(t)$ and U. A positive-valued slope function $\beta_1(t)$ indicates a positive association between $Y(t)$ and $X(t)$ at time t, while a negative-valued slope implies a negative association, and this also applies to the scalar covariates' dynamic associations with the response Y at time t.

In some cases there may be a time lag in the association between the functional response $Y(t)$ and the functional predictor $X(t)$,

$$Y(t) = \beta_0(t) + \beta_1(t)X(t - \Delta) + \beta_2(t)U + \epsilon(t), \tag{1}$$

for $t > \Delta$ where $\Delta \geq 0$ denotes the lag, which is usually unknown.

When one does not have prior knowledge about Δ, its value can be selected by optimizing a data-adaptive criterion, as follows: Let $Y_i(t_j)$ denote the response process of the ith subject observed at the jth time point t_j and $X_i(t_j)$ be the corresponding predictor process observed at the same time point. Let U_i be the baseline covariate associated with the ith subject. Let $\hat{Y}_i^{\Delta}(t_j)$ be the fitted value for $Y_i(t_j)$ obtained after fitting the functional concurrent regression model based on Δ in (1) using all but the ith subject's observations. Then the mean-normalized leave-one-out prediction error $\mathrm{P}_{\mathrm{error}}(\Delta)$ is defined as

$$\mathrm{P}_{\mathrm{error}}(\Delta) = \frac{1}{n}\sum_{i=1}^{n} \frac{||Y_i - \hat{Y}_i^{\Delta}||_{\mathcal{L}_2}}{||Y_i||_{\mathcal{L}_2}}.$$

The normalization factor $||Y_i||_{\mathcal{L}_2}$ is included to balance the magnitude of $||Y_i - \hat{Y}_i^{\Delta}||_{\mathcal{L}_2}$ for situations where the response curves Y_i are on different scales, which is the case in our application. The optimal Δ is chosen as

$$\hat{\Delta} = \underset{\mathcal{I}_\Delta}{\operatorname{argmin}}\, \mathrm{P}_{\mathrm{error}}(\Delta),$$

where $\mathcal{I}_\Delta$ is the set of potential candidates for Δ.

S.4 Historical Functional Linear Model

Prediction of doubling rates is based on the historical functional linear regression model with scalar response Y and functional predictors $C(s), W(s)$, with $t-13 \leq s \leq t-1$,

$$Y = \beta_0 + \int_{t-13}^{t-1} \beta_1(s)C(s)ds + \int_{t-13}^{t-1} \beta_2(s)W(s)ds + \epsilon,$$

where ϵ is a mean-zero error term. The functions $\beta_1(s), \beta_2(s)$ are then estimated by representing them in the eigenfunctions of $C(s), W(s)$ respectively, which are assumed to form a basis of the function space L^2. Writing $\xi_{C,k},\ \phi_{C,k}(s),\ \xi_{W,k},\ \phi_{W,k}(s)$ to denote functional principal component scores and eigenfunctions of $C(s)$ and $W(s)$, and letting

$$\beta_1(s) = \sum_{k=1}^{\infty} \beta_{1,k}\phi_{C,k}(s),$$
$$\beta_2(s) = \sum_{k=1}^{\infty} \beta_{2,k}\phi_{W,k}(s),$$

we truncate the sum at K included terms so that the model becomes

$$\begin{aligned} Y &= \beta_0 + \sum_{k=1}^{K} \beta_{1,k} \int_{t-13}^{t-1} \phi_{C,k}(s)C(s)ds + \sum_{k=1}^{\infty} \beta_{2,k} \int_{t-13}^{t-1} \phi_{W,k}(s)W(s)ds + \epsilon \qquad (2) \\ &= \beta_0 + \sum_{k=1}^{K} \beta_{1,k}\xi_{C,k} + \sum_{k=1}^{K} \beta_{2,k}\xi_{W,k} + \epsilon. \qquad (3) \end{aligned}$$

The $\beta_{1,k}$ can then be estimated by simply fitting a linear regression model with predictors $\xi_{C,k}, \xi_{W,k}$, $k = 1, 2, \ldots, K$. The evaluation of the model is the same as in Section S.3, where we used $R^2(t) = 1 - \frac{\mathrm{var}(\epsilon(t))}{\mathrm{var}(Y(t))}$.

S.5 Empirical Dynamics

The foundation of FDA relies on the assumption that the observed sample of trajectories is generated from an underlying smooth and square integrable process. We also assume that derivatives exist that can then be utilized to study the underlying dynamics of the process [4, 5, 6]. For both functional and longitudinal data, empirical dynamics [7] provides a principled approach to learn and quantify the dynamics. The motivation for empirical dynamics is the fact that for a differentiable Gaussian process $Y(t)$ with mean $\mu(t)$ one

has the decomposition

$$\frac{d}{dt}[Y(t) - \mu(t)] = \beta(t)\,(Y(t) - \mu(t)) + Z(t),$$

where $\beta(t) = \frac{d}{dt}\log[\text{var}\{Y(t)\}]$ is a smooth dynamic coefficient function and $Z(t)$ is a random drift process independent of $Y(t)$. This leads to the linear model formulation

$$E\left(\frac{d}{dt}[Y(t) - \mu(t)]|Y(t)\right) = \beta(t)(Y(t) - \mu(t)) \tag{4}$$

that can be analyzed under the functional concurrent regression framework described in Section S.3. For non-Gaussian processes, model (4) yields effective approximations in the least squares sense, where one can use the coefficient of determination

$$R^2(t) = 1 - \frac{\text{var}(Z(t))}{\text{var}\left(\frac{d}{dt}Y(t)\right)}$$

to gauge the fraction of variance in $\frac{d}{dt}Y(t)$ explained linearly by $Y(t)$. On domains where $R^2(t)$ is large, the linear term $\beta(t)(Y(t) - \mu(t))$ in model (4) plays a significant role in explaining the dynamics of $Y(t)$, while otherwise the random drift process $Z(t)$ becomes the major component.

The coefficient function $\beta(t)$ summarizes the characteristics of the dynamics of the underlying process. If $\beta(t) < 0$, one observes *centripetality* or dynamic regression to the mean. That is to say, a trajectory which lies away from the mean function tends to move closer toward the mean function as time progresses. If on the other hand $\beta(t) > 0$, one has *centrifugality* or dynamic explosive behavior, since deviations from the mean at time t tend to result in further departures from the mean as t increases.

Functional concurrent regression, described in Section S.3, can be used to add additional functional predictors and baseline covariates in model (4) which allows practitioners to study empirical dynamics of the observed trajectories while simultaneously controlling for other predictors (with or without time lags) and baseline covariates. For a functional predictor $X(t)$ and a baseline covariate U, the linear model

$$E\left(\frac{d}{dt}[Y(t)-\mu(t)] \;\Big|\; Y(t)\right) = \beta_1(t)(Y(t)-\mu(t)) + \beta_2(t)X(t-\Delta) + \beta_3(t)U + Z(t) \quad (5)$$

provides a systematic approach to study simultaneously the empirical dynamics of $Y(t)$ and the dependence on other baseline or functional covariates. The lag Δ can either be set to zero for a truly concurrent approach, or may be selected using a data adaptive criterion as described in Section S.3 to model a relationship that involves a time delay.

For analyzing empirical dynamics, one needs to estimate derivatives given the observations $Y_i(t_j)$, with notation borrowed from Section S.3. For our applications, we choose to estimate derivatives by local quadratic smoothing [8, 9] using the Epanechnikov kernel and a bandwidth of 2 days.

S.6 Rank dynamics

For functional data, cross-sectional ranks and their temporal dynamics may be investigated through the rank processes and summary statistics for rank dynamics as per [10]. For a generic stochastic process $Y : \mathcal{T} \to \mathbb{R}$, our starting point is the cross-sectional distribution $P(Y(t) \leq z) =: F_t(z)$, for each $t \in \mathcal{T}$. Without loss of generality, we consider $\mathcal{T} = [0, 1]$. The rank processes S_i associated with trajectories Y_i are then given by

$$S_i(t) = F_t(Y_i(t)). \quad (6)$$

To summarize the overall rank of each trajectory and quantify how each rank trajectory varies with time, we consider two summary measures for rank processes, individual-specific integrated rank ρ_i and rank volatility ν_i, defined as

$$\rho_i = \int_{\mathcal{T}} S_i(t)\mathrm{d}t, \quad \text{and} \quad \nu_i = \int_{\mathcal{T}} (S_i(t) - \rho_i)^2 \mathrm{d}t, \tag{7}$$

respectively. For the COVID-19 data, as described before, the caseload trajectories $C(t)$ are observed on the same time grid $\mathcal{T} = [0, 66]$ for each country. A straightforward estimate for the rank processes in (6) is then given by replacing the cross-sectional distribution function F_t by its empirical counterpart, i.e.,

$$\widehat{S}_i(T_j) = \frac{1}{n} \sum_{l \neq i} \mathbf{1}_{\{Y_l(T_j) \leq Y_i(T_j)\}}. \tag{8}$$

Hence, individual-specific integrated rank ρ_i and rank volatility ν_i in (7) can be estimated by plugging in the estimated rank processes in (8) and taking numerical integration. The results are shown in Figure 2.

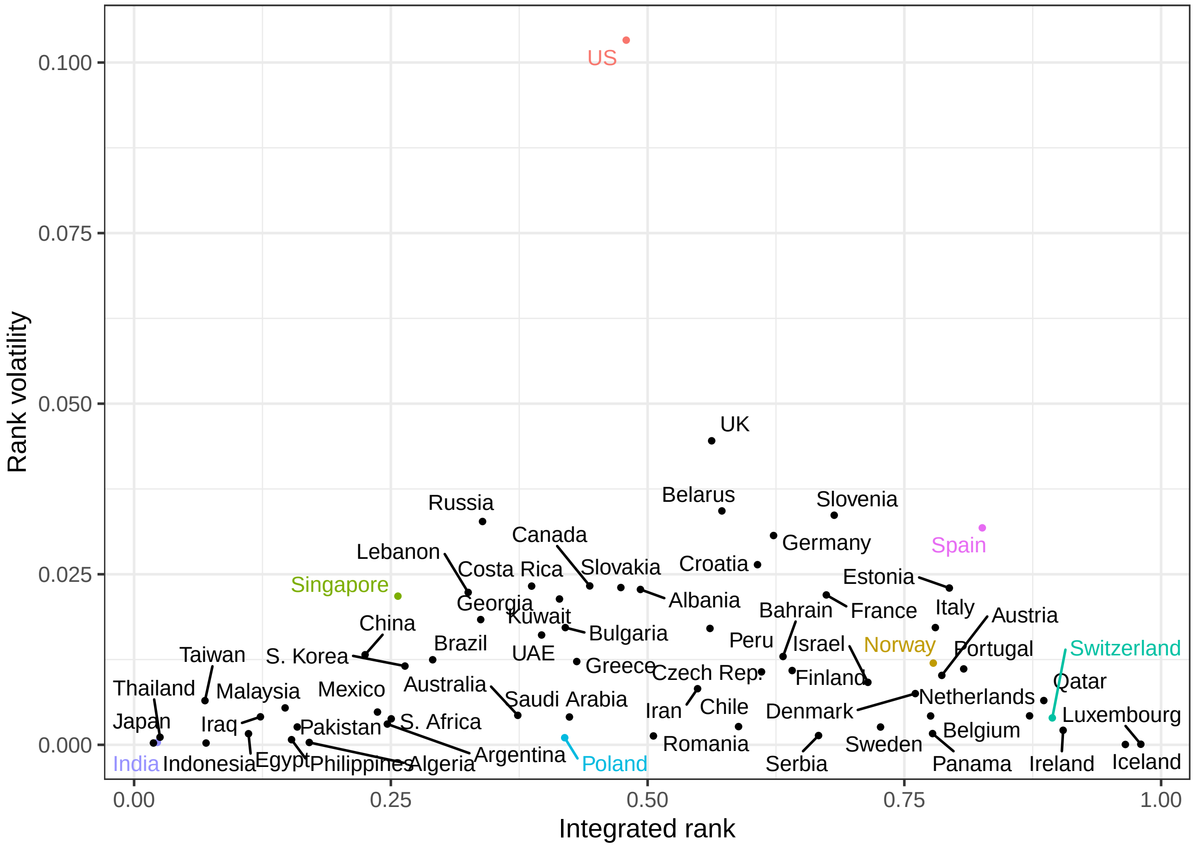

Figure 2: Integrated ranks ρ_i versus rank volatility ν_i as per (7) for empirical ranks during the first 67 days since exposure.

S.7 Mobility Data

Figure 3 and 4 display the trends pertaining to different categories from the Google community mobility data for selected countries.

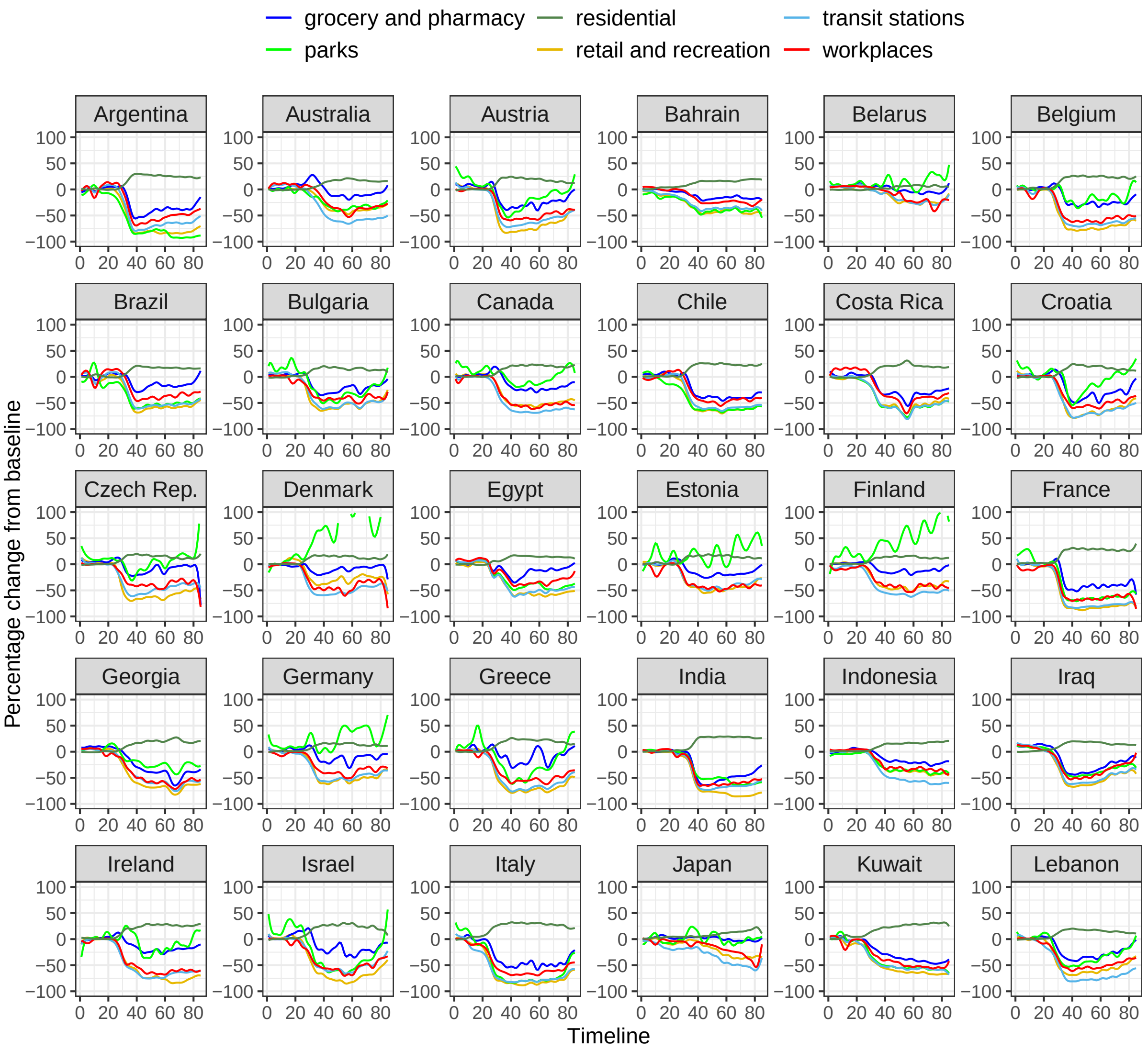

Figure 3: Mobility Patterns: Argentina-Lebanon

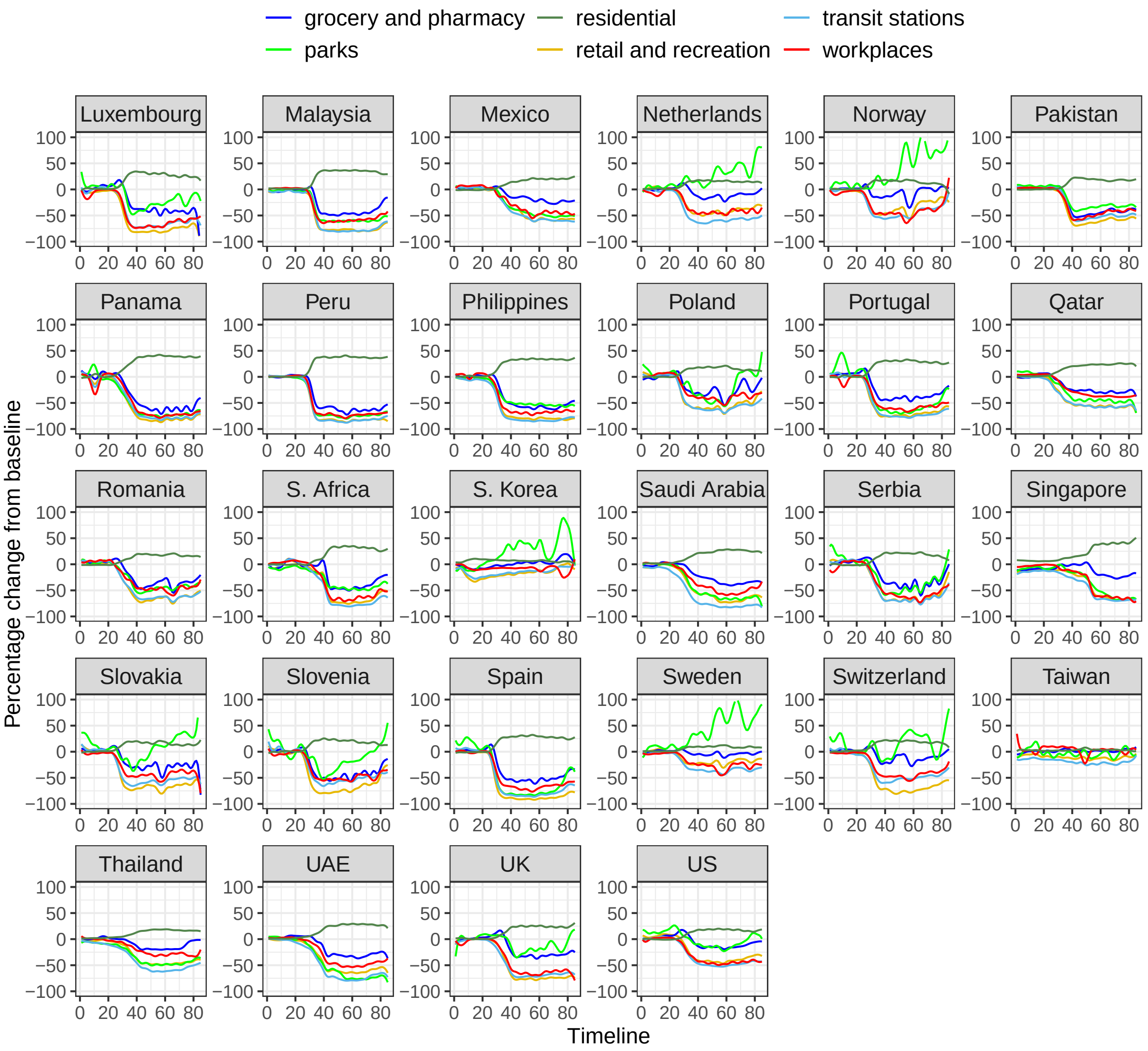

Figure 4: Mobility Patterns: Luxembourg - US

References

[1] Zongwu Cai, Jianqing Fan, and Runze Li. Efficient estimation and inferences for varying-coefficient models. *Journal of the American Statistical Association*, 95(451):888–902, 2000.

[2] Damla Şentürk and Hans-Georg Müller. Functional varying coefficient models for longitudinal data. *Journal of the American Statistical Association*, 105(491):1256–1264, 2010.

[3] Jianhua Z. Huang, Colin O. Wu, and Lan Zhou. Polynomial spline estimation and inference for varying coefficient models with longitudinal data. *Statistica Sinica*, pages 763–788, 2004.

[4] James O. Ramsay. Differential equation models for statistical functions. *Canadian Journal of Statistics*, 28(2):225–240, 2000.

[5] Jim O. Ramsay, Giles Hooker, David Campbell, and Jiguo Cao. Parameter estimation for differential equations: a generalized smoothing approach. *Journal of the Royal Statistical Society: Series B (Statistical Methodology)*, 69(5):741–796, 2007.

[6] André Mas and Besnik Pumo. Functional linear regression with derivatives. *Journal of Nonparametric Statistics*, 21(1):19–40, 2009.

[7] Hans-Georg Müller and Fang Yao. Empirical dynamics for longitudinal data. *The Annals of Statistics*, 38(6):3458–3486, 2010.

[8] Theo Gasser and Hans-Georg Müller. Estimating regression functions and their derivatives by the kernel method. *Scandinavian Journal of Statistics*, pages 171–185, 1984.

[9] Hans-Georg Müller, U. Stadtmüller, and Thomas Schmitt. Bandwidth choice and confidence intervals for derivatives of noisy data. *Biometrika*, 74:743–749, 1987.

[10] Yaqing Chen, Matthew Dawson, and Hans-Georg Müller. Rank dynamics for functional data. *Computational Statistics & Data Analysis*, 2020.
